# Intranodal lymphangiography combined with foam sclerotherapy embolization of thoracic duct in the treatment of postoperative chylous leakage for thyroid carcinoma: a case report and review

**DOI:** 10.3389/fradi.2024.1476227

**Published:** 2024-09-27

**Authors:** RuiJiang Liu, Lei Cao, JingXin Du, Ping Xie

**Affiliations:** ^1^ School of Medical and Life Sciences, Chengdu University of Traditional Chinese Medicine, Chengdu, Sichuan, China; ^2^ Radiology Department, Sichuan Academy of Medical Sciences & Sichuan Provincial People’s Hospital, Chinese Academy of Sciences, Chengdu, Sichuan, China

**Keywords:** chylous leakage, thoracic duct angiography, sclerotherapy, papillary thyroid carcinoma, Interventional Radiology

## Abstract

**Background:**

Chylous leakage (CL) is a rare but significant complication following cervical lymph node dissection, particularly in patients with papillary thyroid carcinoma (PTC). This condition is characterized by the leakage of lymphatic fluid, which can result in severe consequences such as malnutrition, immunosuppression, and prolonged hospital stays. Conventional treatments for CL include conservative measures and surgical interventions, but these approaches often face limitations and challenges. This case report discusses a successful treatment of CL using thoracic duct lymphangiography combined with local injection of sclerotherapy, demonstrating a novel and effective approach for managing this complication.

**Case presentation:**

A 72-year-old female patient with PTC underwent total thyroidectomy and bilateral Level VI and left Levels II, III, IV, and V cervical lymph node dissection. Postoperatively, the patient developed milky drainage indicative of CL. Despite initial conservative treatments including pressure bandaging, negative pressure drainage, and nutritional adjustments, the patient's condition did not improve. The patient declined surgical options, leading to the decision to perform thoracic duct lymphangiography combined with local injection of sclerotherapy. Under real-time ultrasound guidance, the inguinal lymph nodes were accessed, and lipiodol was injected to visualize the thoracic duct. Subsequently, foam sclerosant was injected at the leakage site under fluoroscopic guidance. The procedure resulted in a significant reduction of chyle leakage, and the patient was discharged with no recurrence during a 1-year follow-up.

**Conclusions:**

This case illustrates that thoracic duct angiography combined with local injection of sclerotherapy can be an effective treatment for high-output CL when conservative measures fail and surgical intervention is not preferred. The approach offers a minimally invasive alternative that can reduce complications and improve patient outcomes. The successful management of CL in this case underscores the potential of advanced interventional techniques in treating lymphatic system complications and highlights the need for further research to establish standardized treatment protocols.

## Introduction

The incidence rate of thyroid carcinoma, the most common malignant tumor of the endocrine system, has been increasing in recent years. Currently, thyroid carcinoma cases rank 7th and 4th in the list of malignant tumors diagnosed among the female population in the United States and China, respectively (1–3). Papillary thyroid carcinoma (PTC) is the most common pathological type and has the potential for early lymph node metastasis, which occurs at the lateral cervical region in 30%–80% of the patients (4). Total thyroidectomy and central or lateral cervical dissection are common procedures for treating PTC with lateral cervical lymph node metastasis (5).

Chylous leakage (CL) is a severe complication after cervical lymph node dissection, with an incidence rate of 1%–3%, and usually, it occurs on the left side of the neck (6). The loss of proteins, fats, fat-soluble vitamins, micronutrients, and lymphocytes caused by CL can lead to delayed wound healing, electrolyte disturbances, malnutrition, and immunosoppressions (7). Especially in high output cases, which is defined as greater than 500 ml over 24 h, CL may considerably affect patients’ quality of life after undergoing surgical procedures, prolonging their hospital stay and increasing the cost of treatment (8). Due to the low incidence rate, complicated conditions, and various relevant treatment options, there are still no standardized treatment methods and guidelines for diagnosis and treatment for CL (9, 10). Herein, we present a case of CL after lateral cervical dissection for PTC. After a month of failure with conservative and local treatment, we obtained immediate results by performing thoracic duct angiography with lipiodol combined with local injection of sclerotherapy.

## Case

A 72-year-old female patient with PTC underwent total thyroidectomy and bilateral Level VI and left Levels II, III, IV, and V cervical lymph node dissection. A histological examination showed bilateral thyroid papillary cancer with extrathyroid extension and lateral lymph nodes metastasis. Forty-three lymph nodes were identified with tumor infiltration, especially in the junction of the internal jugular vein and subclavian vein. Milky drainage developed on the second day after surgery and became more severe over the following days. Immediate conservative measures were taken, including pressure bandaging, negative pressure drainage, and nutritional adjustment, aiming to stop the loss of fluid and electrolytes, plasma proteins, fat, and immunomodulatory lymphocytes. After 2 weeks of conservative treatment, the patient still did not respond well. Although the drainage volume had reached 3,230 ml/24 h, the patient refused surgical treatment or thoracoscopic ligation of the thoracic duct recommended by us due to anxiety. On the 19th day after surgery, the patient's drainage volume increased to 4,390 ml. After obtaining her consent, Pseudomonas aeruginosa injection (PAI) was administered to the wound bed under ultrasound guidance. A total of 15 ml PAI was injected on days 20, 23, and 25 after surgery, 5 ml each time. The drainage tube was clamped for at least 30 min after injection, and during this period, chylous was reduced by nearly half.

**Figure 1:**
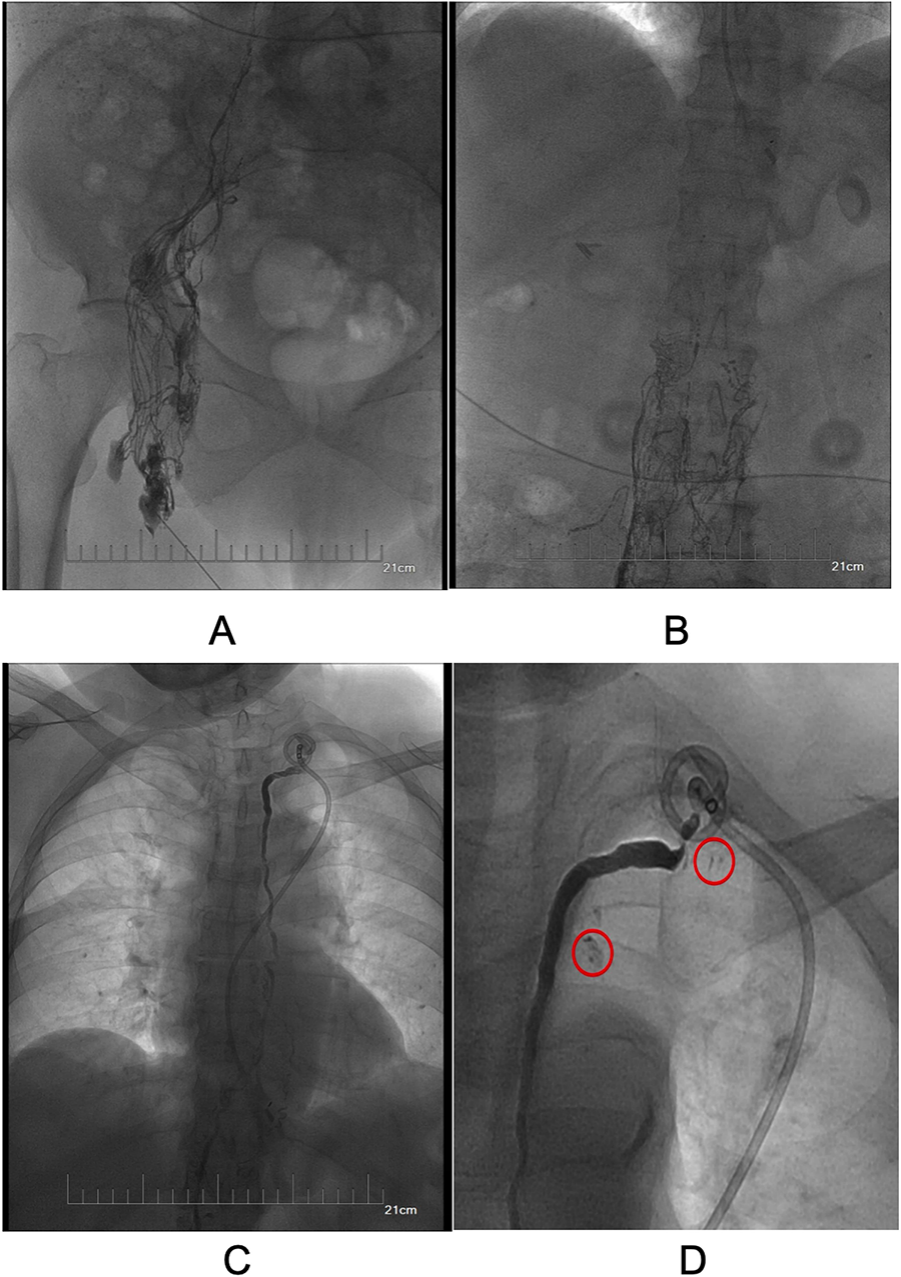
**(A)** Visualized ipsilateral pelvic lymphatic vessels and lymph nodes. **(B)** Visualized cisterna chyli. **(C)** Visualization of the total thoracic duct. **(D)**. Thoracic duct disruption and lipiodol extravasation at the left venous angle (red circles).

**Figure 2:**
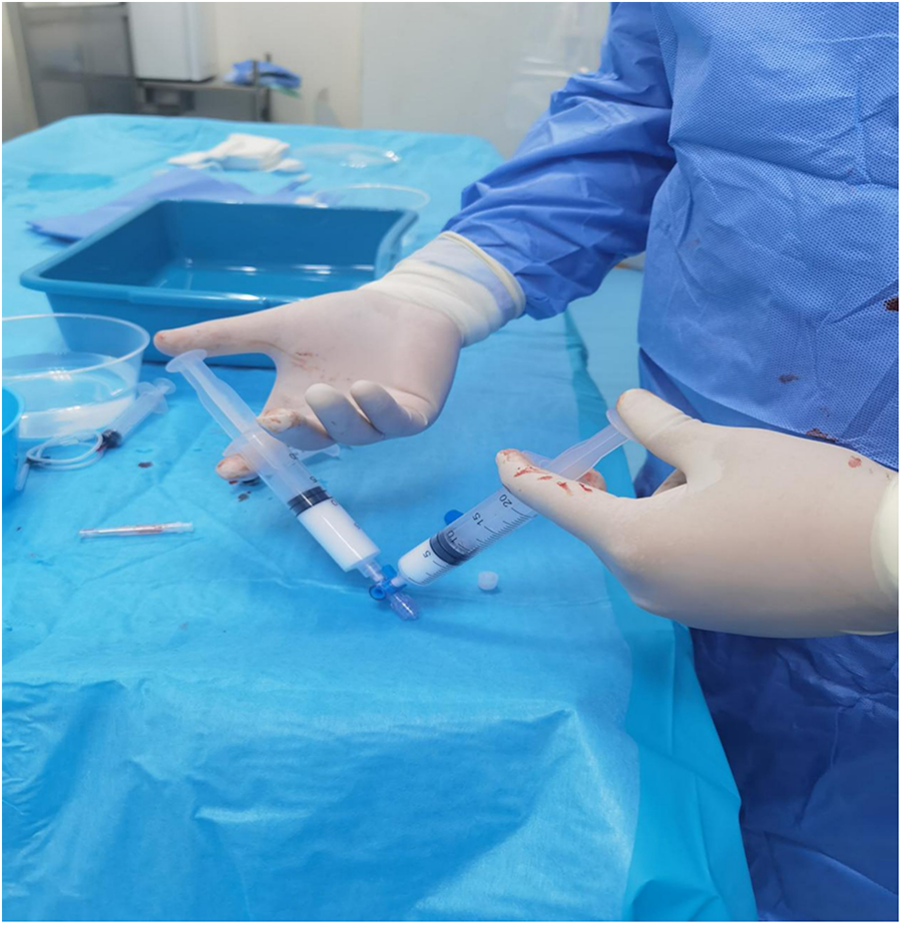
The foam sclerosants was prepared by using two 10 ml empty syringes connected with a three-way valve, in which 1 ml of lauromacrogol and 3 ml of air were extracted, respectively. Then, the piston was quickly pushed to mix the liquid and gas until foam forms.

**Figure 3:**
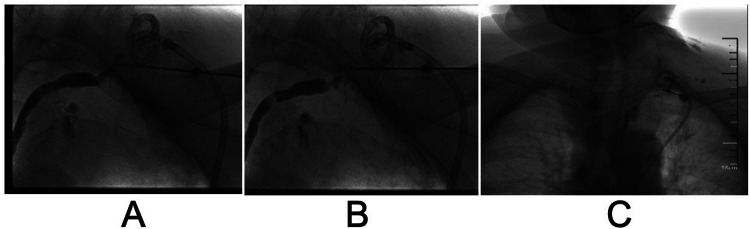
Stepwise reverse flow of foam sclerosant into the thoracic duct step by step until the stagnation of iodized oil. **(A)** Puncture of the end of the thoracic duct through the left venous angle. **(B)** Reverse flow of iodized oil observed at the end of the thoracic duct after injection of foam sclerosant. **(C)** Stagnation of iodized oil at the end of the thoracic duct after injection of foam sclerosant.

However, the drainage returned to 4,220 ml/24 h on day 26 after surgical intervention. The patient still refused surgery, so we proposed thoracic duct angiography combined with local sclerotherapy and performed it on the 35th day after surgery. Upon local anesthesia, the right inguinal lymph nodes were accessed with a 25-gauge needle under real-time ultrasound guidance. A small amount of lipiodol is injected slowly into the lymph node to reveal the efferent lymphatic vessels. Under the real-time monitoring of fluoroscopy, approximately 10 ml of lipiodol was continuously injected into the inguinal lymph node once the contrast opacifies the iliac lymph node (Figure 1A). After approximately 11 min, the lipiodol accumulated in the cisterna chyli (Figure 1B), and after 15 min, the whole thoracic duct could be visualized (Figure 1C). At the same time, thoracic duct disruption and lipiodol extravasation were found near the left venous angle (Figure 1D).Then, the foam sclerosants was prepared by using two 10 ml empty syringes connected with a three-way valve, in which 1 ml of lauromacrogol and 3 ml of air were extracted, respectively. Then, the piston was quickly pushed to mix the liquid and gas until foam forms. After the leak point was located by cone beam CT (CBCT), the end of the thoracic duct was punctured from the neck using a 21-gauge Chiba needle and injected approximate 1 ml of foam sclerosant under fluoroscopy. The operation was stopped after approximately 2 cm of lipiodol reflux was observed (Figures 2, 3). The patient was observed in the operating room for 30 min without lipiodol extravasation. Over the next 3 days, the patient is in stable condition with no chyle from the drainage tube and no swelling in the neck. The patient was discharged after removal of the drainage tube. During the 1-year follow-up, there was no recurrence of chyle leakage or other complications.

## Discussion

CL is a rare complication after cervical lymph node dissection in patients with thyroid cancer, with an incidence of 1%–3% (11). Due to the low incidence, there are no relevant large-scale studies, and this complication will pose a great challenge to patients and physicians once it occurs.

At present, the treatment of CL is not uniform: non-surgical treatment and surgical treatment are the main measures to treat CL after cervical lymph node dissection (11, 12). In addition, topical therapy and TDE as a minimally invasive option for this complication have also been reported (13).

The non-surgical treatment methods include diet control, local compression, negative pressure drainage, somatostatin/somatostatin analogs and local treatment (14, 15). Low-fat or fat-free diets can reduce chyle formation and reduce leakage, but without proper management, patients may suffer from malnutrition, hypoproteinemia, and electrolyte disturbances (12). Local compression can prevent chyle from entering the surrounding tissue spaces, facilitate the closure of lymphatic vessels and promote flap fitting (16). At the cervical region, local compression has a certain effect, but relevant procedures are more complex, and the pressure is not easy to control (12, 17, 18). Negative pressure suction under a certain pressure can not only drain fluid but also promote the occlusion of lymphatic vessels as an effective method for preventing CL (19). However, it is difficult to maintain an appropriate negative pressure, and there is no unified standard for negative pressure. While, there are successful cases reported using −10 kpa negative pressure and −50 to −60 kpa negative pressure (11, 14, 16). After all, lower negative pressure is less attractive and less effective and excessive negative pressure tends to cause flap necrosis, some patients cannot bear the pain caused by high negative pressure, so the pressure has to be reduced (14). Somatostatin analogs reduces the production of lymphatic fluid through endocrine and paracrine systems. It may also indirectly reduce the production of lymphatic fluid by inhibiting pancreatic secretion and lowering gastrointestinal peristalsis, portal vein pressure and visceral blood flow. In the treatment of CL, somatostatin can shorten the healing time, reduce the loss of lymphatic fluid, and promote whole-body rehabilitation (20).

From the results of the literature search, Conservative measures have shown a certain effect in the treatment of CL, especially for low output cases (7, 16, 21). It is the first choice for all patients with chyle leakage because of its advantages of small trauma, less cost and easy popularization. However, the disadvantage is also obvious, that is, the effect is not significant for high output cases (12).

The surgical treatment of CL after cervical lymph node dissection mainly includes surgical exploration and thoracic duct ligation under thoracoscope, which is usually adopted when conservative measures are ineffective (11, 22). In the former, the area of CL (usually the left jugular angle) is exposed through the original surgical incision, and then the thoracic duct is ligated or the venous angle is filled with myocutaneous flap to achieve the purpose of closure. Thoracoscopic thoracic duct ligation is to locate the thoracic duct by thoracoscopic technology and block it, so as to stop cervical CL. From the literature reports, surgical treatment is almost the only treatment for patients with high output or patients who failed non-surgical treatment, but the disadvantages of large trauma, high cost and difficult operation limit its popularity (22, 23). This is also the reason for surgical treatment as a second-line option. Therefore, we need a middle way between surgical treatment and non-surgical treatment to treat CL.

Local treatment and thoracic duct embolization (TDE), as minimally invasive measures between conservative treatment and surgical treatment, seem to be an ideal approach (11–13). Local treatment involves injection of drugs such as anhydrous alcohol, doxycycline, hypertonic glucose injection, pseudomonas aeruginosa injection or sclerosing agents into the wound, usually at the left jugular Angle, to treat CL. Yamaki et al. (24) and Mahrer et al. (25) successively reported foam sclerosant used for the treatment of lymphatic malformation. Subsequently, Kortes et al. (26) and Pan et al. (27) proposed a new scheme for afferent lymphatic vessel sclerotherapy (ALVS) guided by CT, and applied the method to patients with lymphatic leakage who did not respond to non-surgical treatment. The success rate of ALVS is as high as 88.9%. In China, Hao et al. (10) achieved good results in the treatment of low output CL with 50% glucose injection and Pingyangmycin local injection. In their study, the effect of patients with CL greater than 450 ml/24 h was poor, and finally surgical treatment was performed. Pseudomonas aeruginosa injection, a biological agent covered with fimbriae, can be obtained with the use of genetic recombination technology. The fimbriae of pseudomonas aeruginosa are glycoprotein ligands of high adhesion. When postoperative subcutaneous effusion occurs, pseudomonas aeruginosa injection administered subcutaneously and in or around the residual cavity may cause local aseptic inflammation, thus promoting the adhesion of skin and muscle wounds and accelerating the closure of lymphatic vessels for wound healing (19). Wei et al. (28) adopted the use of pseudomonas aeruginosa injection in the treatment of CL after cervical lymph node dissection, and achieved good results. Mei et al. (29) successfully treated two cases of complex CL with PAI, one of which had a high output CL (maximum drainage volume: 1,170 ml/24 h, and this patient achieved success with local application of PAI after failed reparative treatment. It can be seen that for low output chyle leakage, local treatment has shown good efficacy. However, the effectiveness of local treatment in patients with high output has not been widely reported.

Thoracic duct embolization (TDE) is an interventional technique. Cope et al. (30) proposed a groundbreaking method based on the percutaneous catheterization and embolization of the thoracic duct guided by lymphangiography for treating chylothorax in 1998. TDE consists of three steps. First, the thoracic duct is visualized by injecting lipiodol into the lymphatic vessels via a transpedal access. The second step was thoracic duct catheterization via a transabdominal approach, and the third step was to insert embolization materials through the catheter to embolize the thoracic duct. Most European and American scholars represented by the Clinic for Diagnostic and Interventional Radiology of the University Hospital Heidelberg have performed TDE using embolization materials such as anhydrous alcohol or coil. In the report by Cope et al., TDE was significantly effective in treating CL (31). Compared with conventional surgical methods, TDE has a high success rate and low incidence rate of postoperative complications. In the past 20 years, it has gradually replaced thoracic duct ligation as a secondary regimen for patients with lymphatic leakage after thyroidectomy carcinoma when conservative treatment fails (26, 27, 32, 33). The deficiency of TDE is mainly reflected in the technical difficulty. It is not easy to puncture the thoracic duct via a transabdominal approach. Itkin et al. described the procedure for TDE in detail and found that the key to controlling leakage was successful catheterization of the thoracic duct. Although catheterization fails in more than 30% of patients, the cure rate of CL can reach 90% once the catheter is successfully inserted into the thoracic duct (34). There are also some uncertainties regarding the embolic material. There is also some uncertainty about the embolization material, the use of anhydrous alcohol may result in a large embolization range due to easy flow, whereas coils can embolize only a smaller range, which may increase the risk of tissue damage or recurrence of lymphatic leakage. In addition, lymphangiography via transpedal access would be time-consuming, which limits the popularity of TDE.

In recent years, intranodal lymphangiography has been popularized in different medical communities worldwide. Compared with conventional pedal lymphangiography, intranodal lymphangiography has the advantages of simplicity, less trauma and shorter time consuming (35, 36). It has profoundly impacted the development of lymphangiography techniques and interventional therapies for the lymphatic system diseases. Intranodal lymphangiography is usually performed by direct puncture of inguinal lymph nodes guided by an ultrasound probe of frequency as high as >7.5 MHz. Generally, a large lymph node is selected, and the needle tip is placed in the transitional region between the cortex and the hilum of the lymph node. Subsequently, under intermittent fluoroscopy, lipiodol is manually injected at a rate as slow as 1–2 ml/5 min, allowing the clinician to observe the ascending speed and position of the iodized oil and the position of the cisterna chyli. Usually, the thoracic duct can be visualized 40 min after the procedure. In addition to the diagnostic value, lymphangiography combined with interventional therapy has a good therapeutic effect on CL, according to relevant literature (26, 37).

In this case, CL occurred on the second day after surgery with a large amount of leakage, which was most likely caused by edema at the thoracic duct ligation and high local reflux pressure, which ultimately led to the rupture of the main thoracic duct. For such a patient with a maximum leakage close to 5,000 ml/24 h, conservative treatment is obviously ineffective. Local treatment with PAI has also failed. After rejecting both surgical exploration and thoracoscopic thoracic duct ligation, the patient agreed to receive TDE. Under ultrasound guidance, the inguinal lymph nodes were punctured, followed by the injection of lipiodol. Real-time fluoroscopic monitoring demonstrated the gradual opacification of the thoracic duct from caudal to cranial direction over a duration of approximately 20 min, culminating in leakage at the cervical region. As the procedure progressed, it was observed that the flow rate of lipiodol slowed, and the accumulation of lipiodol at the leakage site did not significantly increase; however, a slight reduction in the drainage of milky chylous fluid was noted. Therefore, based on the methods proposed by Yamaki et al. (24) and Markovic et al. (38) for the treatment of vascular malformations. After discussion and with the consent of the patient, we replaced thoracic duct embolism with local injection of foam sclerosant to reduce the potential risk and complications of thoracic catheter catheterization. Finally, this combination achieved immediate results.

The effective mechanism of this measure can be concluded as follows: sclerosing foam can be injected directly around leak point, which was precisely located by angiography. Lauromacrogol induces local aseptic inflammation, loss of secretory function, regional adhesions, and fibrosis, causing occlusion of lymphatic vessels and terminating the leakage (Figure 2). The foam can fill the wound cavity, increase the contact area between drugs and surrounding tissues, and has small mobility and long local residence time, which can reduce its dose and reduce the occurrence of related complications. The thick lipiodol slows the flow of lymph in the thoracic duct, lipiodol do not dilute quickly, allowing adjacent tissues to adequately respond to inflammation.

Lipiodol, a highly viscous iodinated poppy-seed oil, not only acts as a contrast agent in lymphatic system embolization but also has therapeutic effects. Lipiodol can seal small lymphatic leaks and induce local sterile inflammation, promoting tissue repair (39–41).

In cases of extensive lymphatic vessel injury, using a single embolic agent may not be sufficient to fully seal the leakage sites. The combined use of Lipiodol, adjunctive glue embolization, and other embolic agents can effectively reduce the leakage rate, allowing the residual fluid to be absorbed by the body cavity and facilitating the spontaneous healing of damaged lymphatic channels. These techniques have complementary mechanisms, which significantly enhance the treatment outcomes. Furthermore, in the management of postoperative lymphatic leakage, an aggressive interventional approach is recommended when leakage is detected on lymphangiography. The combination of Lipiodol with other embolic agents can help reduce hospital stay and mitigate complications associated with lymphatic leakage (42, 43).

Foam sclerotherapy has shown effective results in treating chylous leakage within the lymphatic system. However, several potential complications need attention, though they are generally mild and manageable. Firstly, foam sclerotherapy can cause over-embolization, obstructing normal lymphatic drainage and leading to localized lymphedema (44, 45). Secondly, there is a risk of inadvertent vascular injection, which could result in thrombophlebitis or other vascular-related issues (46). While these complications are rare, careful monitoring is required to prevent long-term effects. Thus, when administered with precision, foam sclerotherapy remains an effective treatment for chylous leakage.

In this process, we believe that there are two points that need to be paid attention to reduce the occurrence of serious complications, local injection of foam sclerosant must be performed under real-time monitoring of fluoroscopy to avoid direct vascular injury or foam injection into the blood vessels.

## Conclusion

AT all, the successfully treated by lymphangiography combined with interventional embolization for chylous leakage has opened a new door for the diagnosis and treatment of lymphatic diseases, widening the possibilities inside the medical field. For example, in areas rich in lymphatic vessels like the groin, pelvis, retroperitoneum, and axilla, postoperative lymphatic leakage can be quickly and effectively treated by lymphatic embolization. The continuous progress of lymphangiography and interventional techniques and the positive results of related in-depth clinical studies shows that this technique is promising and can bring impactful changes to the medical community.

## Data Availability

The original contributions presented in the study are included in the article/Supplementary Material, further inquiries can be directed to the corresponding author.
